# The Eag Domain Regulates the Voltage-Dependent Inactivation of Rat Eag1 K^+^ Channels

**DOI:** 10.1371/journal.pone.0110423

**Published:** 2014-10-21

**Authors:** Ting-Feng Lin, Guey-Mei Jow, Hsin-Yu Fang, Ssu-Ju Fu, Hao-Han Wu, Mei-Miao Chiu, Chung-Jiuan Jeng

**Affiliations:** 1 Institute of Anatomy and Cell Biology, School of Medicine, National Yang-Ming University, Taipei, Taiwan; 2 Brain Research Center, National Yang-Ming University, Taipei, Taiwan; 3 School of Medicine, Fu-Jen Catholic University, Hsin-Chuang, New Taipei City, Taiwan; University of Waterloo, Canada

## Abstract

Eag (Kv10) and Erg (Kv11) belong to two distinct subfamilies of the ether-à-go-go K^+^ channel family (KCNH). While Erg channels are characterized by an inward-rectifying current-voltage relationship that results from a C-type inactivation, mammalian Eag channels display little or no voltage-dependent inactivation. Although the amino (N)-terminal region such as the eag domain is not required for the C-type inactivation of Erg channels, an N-terminal deletion in mouse Eag1 has been shown to produce a voltage-dependent inactivation. To further discern the role of the eag domain in the inactivation of Eag1 channels, we generated N-terminal chimeras between rat Eag (rEag1) and human Erg (hERG1) channels that involved swapping the eag domain alone or the complete cytoplasmic N-terminal region. Functional analyses indicated that introduction of the homologous hERG1 eag domain led to both a fast phase and a slow phase of channel inactivation in the rEag1 chimeras. By contrast, the inactivation features were retained in the reverse hERG1 chimeras. Furthermore, an eag domain-lacking rEag1 deletion mutant also showed the fast phase of inactivation that was notably attenuated upon co-expression with the rEag1 eag domain fragment, but not with the hERG1 eag domain fragment. Additionally, we have identified a point mutation in the S4–S5 linker region of rEag1 that resulted in a similar inactivation phenotype. Biophysical analyses of these mutant constructs suggested that the inactivation gating of rEag1 was distinctly different from that of hERG1. Overall, our findings are consistent with the notion that the eag domain plays a critical role in regulating the inactivation gating of rEag1. We propose that the eag domain may destabilize or mask an inherent voltage-dependent inactivation of rEag1 K^+^ channels.

## Introduction

The *ether-à-go-go* family (KCNH) of voltage-gated K^+^ channels comprises three gene subfamilies: *eag* (K_V_10), *erg* (*eag*-related gene) (K_V_11), and *elk* (*eag*-like K^+^ channel) (K_V_12) [1]. In mammals, Eag encodes neuron-specific K^+^ channels that are expressed in various regions of the brain [2], [3], [4], [5], whilst Erg K^+^ channels are expressed in a wide range of tissues including the heart and the brain [6]. Despite their abundant expression in the brain, the neurophysiological significance of mammalian Eag K^+^ channels remains elusive. By contrast, human Erg (hERG1) K^+^ channels play a critical role in the membrane repolarization of heart muscles and have been clearly associated with both the inherited and the drug-induced forms of cardiac arrhythmia [7], [8], [9].

The gating property of hERG1 K^+^ channels is characterized by an inward-rectifying current-voltage (I–V) relationship that results from a C-type inactivation, since the inactivation process was neither abolished by the deletion of the amino (N−) terminus nor sensitive to the internal application of tetraethylammonium [10], [11], [12]. In addition, the hERG1 inactivation was significantly affected by pore mutations critical for C-type inactivation in Shaker [10], [11], [13]. Compared to the C-type inactivation of the majority of Shaker constructs [14], however, the hERG1 inactivation is fast and voltage-dependent [10], [11]. Moreover, the recovery from the hERG1 inactivated state, which is also fast and voltage-dependent [11], [12], predominantly entails a direct transition to the open state [11], a process that is exemplified by the presence of a prominent rising phase in the tail current. This tail current shape of hERG1 is also known as the hooked tail current [15].

By contrast, mammalian Eag K^+^ channels display little or no voltage-dependent inactivation [3], [4], [5], [16], [17], [18], [19]. Interestingly, pharmacological manipulation, as well as a mutation in the S6 segment, accelerated and accentuated an otherwise subtle (<10% reduction in current amplitude) form of slow inactivation in human Eag isoform 1 (Eag1) channels [20]. In addition, introduction of homologous hERG1 sequences into the pore-S6 region of bovine or murine Eag1 conferred the hERG1-like inactivation [21], [22], [23]. Conversely, reverse mutations by transplanting Eag1 residues into the pore-S6 region of hERG1 generated the non-inactivating Eag1 phenotype [21], [22], [23]. Together these results are consistent with the notion that the hERG1 C-type inactivation is determined by key residues in the pore-S6 region.

The N-terminal region of all members of the *ether-à-go-go* K^+^ channel family contains the cap sequence and the PAS domain that are collectively known as the eag domain [24], [25], [26], [27], [28]. In addition, the N-terminal region contains an N-linker region that connects the eag domain with the transmembrane S1 segment, with the Eag N-linker region being about 190 amino acids shorter than its counterpart in Erg. Although the N-terminus is not required for the C-type inactivation of hERG1 K^+^ channels, deletion of the eag domain resulted in the deceleration of inactivation kinetics and the lessening of steady-state inactivation [29], [30]. Surprisingly, partial deletion of the cap sequence in rat Eag1 (rEag1) channels gave rise to pseudo-inwardly rectifying currents that remotely resembled the current characteristics of hERG1 [31]. Moreover, an N-terminal deletion that includes the complete eag domain and the majority of the adjacent N-linker region led to a prominent hERG1-like I–V relationship in mouse Eag1 [32].

It is still unclear whether the strong inactivation observed in the Eag1 mutants with N-terminal deletion is merely a mutation-induced form of inactivation, or rather an intrinsic gating process unmasked by the removal of the eag domain. The aim of this study is therefore to delineate the role of the eag domain in the inactivation of Eag1. We have constructed two rEag1 chimeras in which the eag domain was replaced by the homologous sequences from hERG1. Despite the presence of numerous identical amino acid sequences in both the hERG1 and the rEag1 eag domains, these two rEag1 chimeras displayed significant voltage-dependent inactivation. Furthermore, an eag domain-lacking rEag1 deletion mutant also showed prominent inactivation features that were notably attenuated upon co-expression with the rEag1 eag domain, but not with the hERG1 eag domain. Finally, we have identified a point mutation in the S4–S5 linker region of rEag1 that resulted in a similar inactivation phenotype even in the presence of an intact eag domain. Our biophysical analyses of these mutant constructs suggest that the role of the rEag1 eag domain in voltage-dependent inactivation may be distinctly different from that of its counterpart in hERG1.

## Materials and Methods

### Molecular biology

rEag1 (kindly provided by Dr. Olaf Pongs, Institute fur Neurale, Signalverarbeitung, Zentrum fur Molekulare Neurobiologie, Germany) and hERG1 (hERG1a; kindly provided by Dr. Gail A. Robertson, Department of Neuroscience, University of Wisconsin, USA) cDNAs were subcloned into the pcDNA3-myc (Invitrogen) and the pSP64-polyA vectors, respectively. To generate N-terminal chimeras between rEag1 and hERG1, compatible restriction sites were introduced in both rEag1 and hERG1 through silent or missense mutations by using the QuickChange site-directed mutagenesis kit (Stratagene). Chimera P: *Hind*III (in vectors) and *Pme*I#1 (rEag1-A135A & K137K; hERG1-K135F, D136K, & M137L). Chimera N: *Hind*III and *Pme*I#2 (rEag1-V215V, F216F, K217K, & T218L; hERG1-A408L). Eag-domain deletion (Δeag): double-digestion with *Hind*III and *Pme*I#1, followed by insertion of the tri-amino-acid linker sequence LAG. To generate the rEag1 and the hERG1 eag-domain fragments, a stop codon was introduced at the end of the eag domain in rEag1-WT and rEag1-chimera N, respectively. The rEag1 point mutations Y344C, Y344A, and Y344F were also generated by using the QuickChange site-directed mutagenesis kit. All constructs were subject to DNA sequencing verification.

### cRNA preparation and injection into *Xenopus* oocytes

For *in* *vitro* transcription, rEag1 and hERG1 cDNA was linearized with *Xba*I and *EcoR*I, respectively. Capped cRNA was transcribed *in* *vitro* from the linearized cDNA template with the mMessage mMachine T7 kit (Ambion). Concentration of cRNA was determined by gel electrophoresis and verified with spectrophotometry.

Adult female *Xenopus laevis* (African Xenopus Facility, Knysna, South Africa) were anesthetized by immersion in Tricaine (1.5 g/l). All procedures were in accordance with the Guidelines for the Care and Use of Mammals in Neuroscience and Behavioral Research (National Research Council 2003) and approved by the Institutional Animal Care and Use Committee (IACUC) of National Yang-Ming University. Ovarian follicles were removed from *Xenopus* frogs, cut into small pieces, and incubated in ND96 solution [(in mM) 96 NaCl, 2 KCl, 1.8 MgCl_2_, 1.8 CaCl_2_, and 5 HEPES, pH 7.5]. To remove the follicular membrane, *Xenopus* oocytes were incubated in Ca^2+^-free ND96 containing collagenase (2 mg/ml) on an orbital shaker (∼200 rpm) for about 60–90 min at room temperature. After several washes with collagenase-free, Ca^2+^-free ND96, oocytes were transferred to ND96. Stage V-VI oocytes were then selected for cRNA injection. Injected oocytes were stored at 16°C in ND96 solution supplemented with 50 mg/L gentamycin. The total volume of cRNA injection was always 41.4 nl per oocyte. cRNA concentrations from 0.1 up to about 3 µg/µl were used for oocyte injection (*i.e.*, from 4.14 up to 124.2 ng cRNA was injected into an oocyte).

### Two-electrode voltage clamp recording in *Xenopus* oocytes

2–4 days after cRNA injection, oocytes were functionally assayed in a recording bath containing Ringer solution [(in mM): 115 NaCl, 3 KCl, 1.8 CaCl_2_, 10 HEPES, pH 7.2]. Where indicated, 60 KCl was employed (by replacing NaCl) to record tail currents. Niflumic acid (0.5 mM) was added to the bath to minimize the contribution of endogenous Ca^2+^-activated Cl^−^ currents. The bath volume was about 200 µl. An agarose bridge was used to connect the bath solution with a ground chamber (containing 3 M KCl) into which two ground electrodes were inserted. Borosilicate electrodes (0.1–1 MΩ) were filled with 3 M KCl. K^+^ currents through rEag1 or hERG1 channels were acquired with the conventional two-electrode voltage-clamp technique with an OC-725C oocyte clamp (Warner). Data were filtered at 1 kHz (OC-725C oocyte clamp) and digitized at 100 µs per point (10 kHz) using the Digidata 1332A/pCLAMP 8.2 data acquisition system (Molecular Devices). All recordings were performed at room temperature (20–22°C). For ionic current recordings, no leak subtraction for passive membrane properties was performed. To prevent voltage clamp errors due to excessive current amplitudes, for all rEag1 constructs, only data from K^+^ channels with current amplitudes (at +60 mV) of about 5–15 µA were selected for further analyses.

Data analyses were performed via built-in analytical functions of the pCLAMP 8.2 software. Steady-state voltage-dependent gating properties of various rEag1 and hERG1 constructs were studied by the following protocols. For the majority of rEag1 channels, reversal potentials were determined from I–V curves, and steady-state current amplitudes were used to calculate channel conductances at different membrane potentials, which in turn were normalized to the maximum amplitude to obtain the relative fraction of open channels (relative Po). Alternatively, for hErg1 channels as well as rEag1-chimera P and N, isochronal tail currents were normalized to the maximum amplitude to obtain corresponding relative Po values. Data points of non-inactivating channels were fit with a Boltzmann function: Po(V) = 1/{1+exp[(V_0.5a_−V)/*k_a_*]}, where V_0.5a_ is the half-maximal voltage for activation, and *k_a_* the slope factor of the relative Po-V curve. For channels showing significant voltage-dependent inactivation, the relative Po-V curve was fit with two Boltzmann functions: Po(V) = 〈1/{1+exp[(V_0.5a_−V)/*k_a_*]}〉×〈(1−*b*)/{1+exp[(V−V_0.5i_)/*k_i_*]}+*b*〉, where V_0.5a_ and V_0.5i_ represent the half-maximal voltage for activation and inactivation, respectively; *k_a_* and *k_i_* the slope factor for activation and inactivation, respectively; and *b* the non-inactivating relative Po at depolarized potentials [32].

### Cell culture and immunoblotting

Human embryonic kidney (HEK293 T) cells were maintained in Dulbecco’s modified Eagle’s medium (DMEM) supplemented with 2 mM L-glutamine, 100 units/ml penicillin/streptomycin, and 10% (v/v) fetal bovine serum (Hyclone). Cells were maintained at 37°C in a 95% air and 5% CO_2_ humidified incubator and passaged about every four days. Transient transfection was performed by the standard calcium phosphate method. 3 µg of cDNA was added to each well on a 6-well cell culture plate. Two days after transfection, cells were processed for biochemical experiments.

Transfected HEK293T cells were solubilized in ice-cold lysis buffer (20 mM Tris-HCl, pH 7.4, 150 mM NaCl, 10 mM Na_2_HPO_4_, 1% Triton X-100, 0.5% Na-deoxycholate, 0.1% SDS, 1 mM EDTA, and 1 mM phenylmethylsulfonyl fluoride) containing protease inhibitor cocktail (Roche Applied Science). Insolubilized materials were removed by centrifugation. Proteins in cell lysates were separated on 7.5% SDS-PAGE, transferred to nitrocellulose membranes, and detected using mouse anti-myc (clone 9E10) or anti-βactin (1∶10,000; Sigma) antibodies. Blots were then exposed to horseradish peroxidase-conjugated anti-mouse or anti-rabbit IgG (1∶5000; Jackson Immunoresearch Lab), and revealed by an enhanced chemiluminescence detection system (WesternBright ECL, Advansta). Results shown are representative of at least three independent experiments. β-actin was used as a loading control.

### Statistical analyses

All values were presented as mean ± SEM. The significance of the difference between two means was tested using the Student’s *t* test, whereas means from multiple groups were compared using the one-way ANOVA analysis. All statistical analyses were performed with the Origin 7.0 software (Microcal Software).

## Results

### rEag1 chimeras containing the hERG1 eag domain display channel inactivation

**Figure 1:**
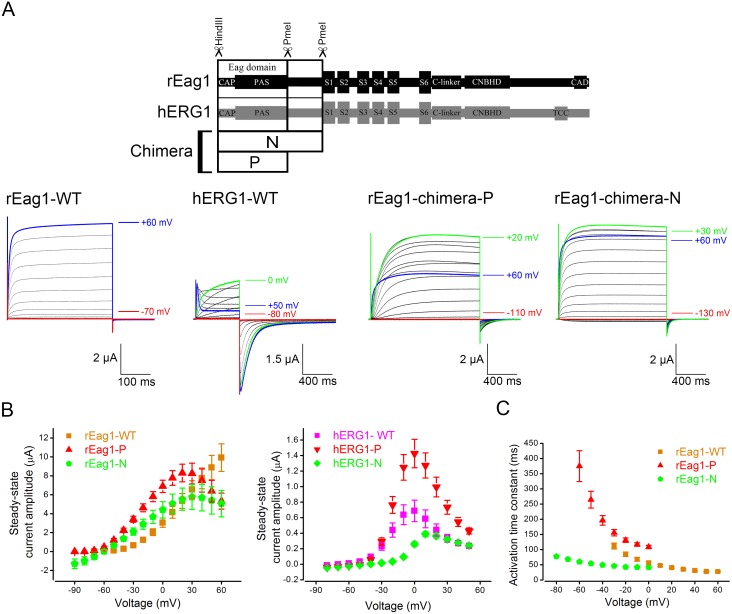
Voltage-dependent inactivation of rEag1 N-terminal chimeras. **(*A*)** (*Top*) Schematic representation of the construction of N-terminal chimeras (see Methods for more detail). (*Bottom*) Representative K^+^ current traces recorded from oocytes expressing rEag1-WT, hERG1-WT, rEag1-chimera P, or rEag1-chimera N channels. The bath solution contained 3 mM KCl. Depending on the steady-state voltage dependence properties of different constructs, the holding potential was set at −90, −110, or −130 mV. The pulse protocol comprised depolarizing test pulses (in 10-mV increments) up to +60 mV, followed by a tail potential at −90 (rEag1-WT), −100 (hERG1-WT), −110 (rEag1-chimera P), or −130 (rEag1-chimera N) mV. *(* ***B*** *)* Steady-state I–V curves in response the test pulses for rEag1 (*left*) and hERG1 (*right*) N-terminal chimeras (*n* = 8–19). *(* ***C*** *)* Activation kinetics of rEag1-WT and N-terminal chimeras. Activation time constants (*n* = 3–6) at indicated potentials were obtained from single exponential fits to the late rising phase of rEag1 currents.

To investigate the potential role of the eag domain in Eag1 inactivation, we focused on two rEag1 chimeric constructs that contained the hERG1 eag domain: chimera P involved swapping the eag domain only, whereas chimera N was constructed by exchanging the complete N-terminus (Fig. 1A) (see Methods for more detail). As illustrated in Figure 1A, both rEag1-chimera P and N produced functional K^+^ channels. Surprisingly, upon strong depolarization to the membrane potential of about +20 mV or higher, both chimeras displayed prominent channel inactivation with hERG1-like I–V relationships (Fig. 1A–B). In rEag1-chimera P, for example, the steady-state current amplitude at +60 mV was notably smaller than that at +20 mV. In addition, compared to rEag1-WT, rEag1-chimera P showed decelerated activation kinetics (Fig. 1C). On the other hand, replacement with the rEag1 eag domain failed to abolish C-type inactivation in the reverse hERG1 chimeras: the inactivation kinetics of hERG1-chimera P was virtually identical with that of hERG1-WT, and hERG1-chimera N exhibited slower but significant inactivation phenotype (Fig. 1B; Fig. S1).

**Figure 2:**
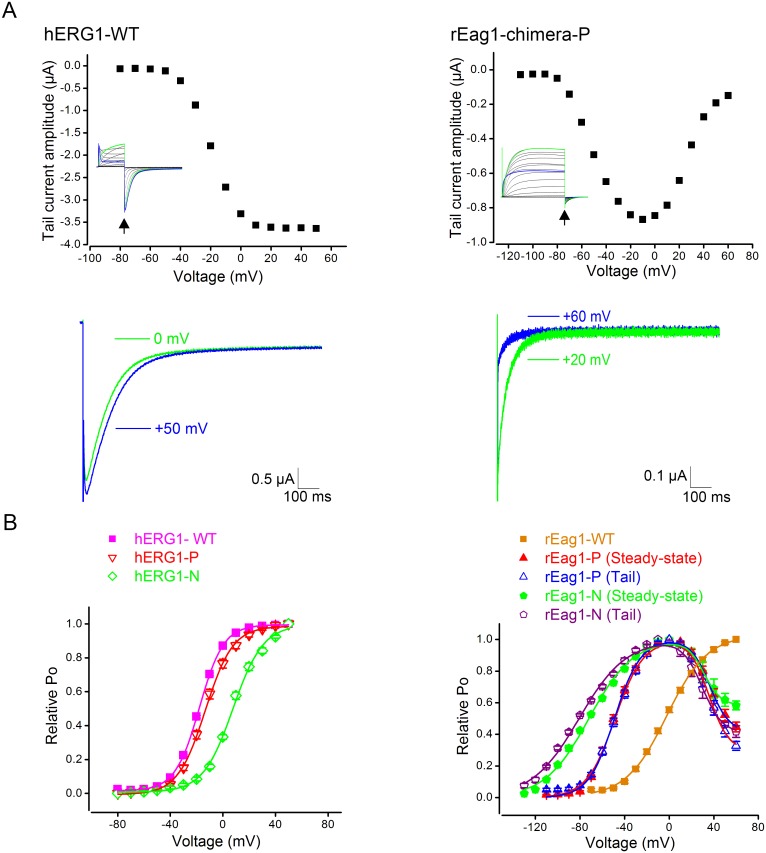
Voltage-dependent reduction in tail current amplitudes for rEag1 N-terminal chimeras. (***A***) (*Top*) Peak tail current amplitudes (*arrows*) for hERG1-WT (*top left*) and rEag1-chimera P (*top right*) current traces shown in Figure 1A. Unlike hERG1-WT, rEag1-chimera P displayed a U-shaped tail I–V curve. (*Bottom*) Comparison of tail current traces induced by two different test pulses for hERG1-WT (*bottom left*) and rEag1-chimera P (*bottom right*). See Figure S2 for more tail current traces of rEag1-chimera P. *(* ***B*** *)* Steady-state activation curves of various hERG1 (*left*) and rEag1 (*right*) constructs. The relative Po was plotted against the corresponding test potential. All data were recorded with 3 mM external KCl. For hERG1 channels, isochronal tail currents were normalized to the corresponding maximum amplitude to obtain relative Po-V curves. For rEag1 channels, steady-state current amplitudes were employed for analyses. For the two rEag1 N-terminal chimeras, tail currents were also used to generate relative Po curves. Data points were fit with one or two Boltzmann equations (*solid curves*). See Methods and Table 1 for more detail.

Despite the presence of hERG1-like I–V relationship, neither of the rEag1 chimeras produced hERG1-like inward tail currents. Upon the application of the tail potential, inactivated hERG1 channels instantaneously recovered back to the open state and then slowly returned to the closed state, manifesting prominent inward tail currents (Fig. 2A), as well as a non-inactivating tail I–V curve that can be transformed into the steady-state activation curve (Fig. 2A–B). The inward tail currents of rEag1 chimera P and N, by contrast, were dramatically smaller (Fig. 1A). Moreover, as the test pulse potential increased to about −10 mV or higher, the peak tail current amplitude of rEag1-chimera P, for example, became progressively smaller, resulting in a U-shaped tail I–V curve that resembled a mirror image of its inactivating steady-state I–V curve (Fig. 2A–B). This U-shaped tail I–V curve is indicative of a voltage-dependent, direct transition from the inactivated state to the closed state. A similar tail current phenotype was also observed for rEag1-chimera N (Fig. 2B). The presence of the rEag1 eag domain, nevertheless, did not abrogate the prominent inward tail currents in the reverse hERG1 chimeras (Fig. 2B; Fig. S1).

**Figure 3:**
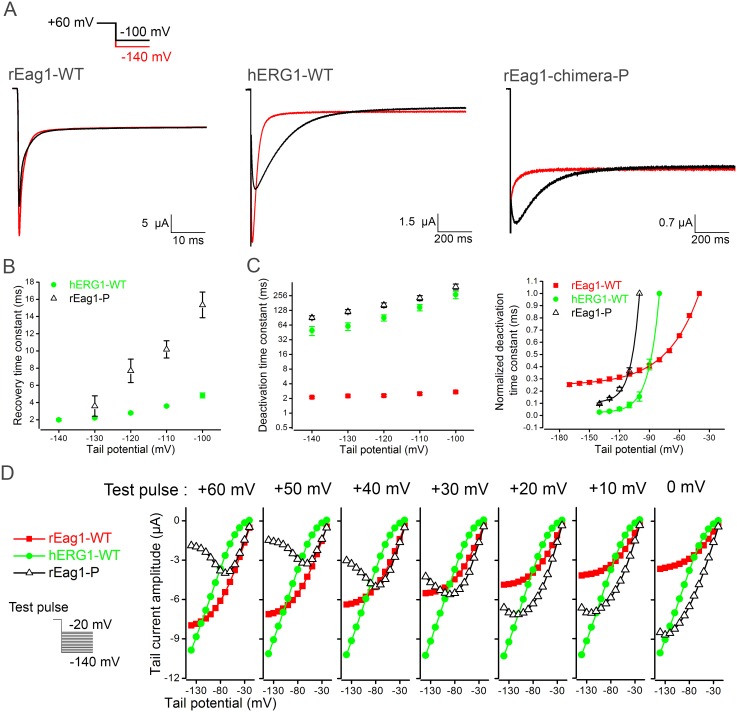
Inactivated rEag1-chimera P channels return to both the open state and the closed state. All data were recorded in 60 mM KCl bath solution. (***A***) Comparison of tail current traces for rEag1-WT, hERG1-WT, and rEag1-chimera P. Channels were subject to a +60 mV test pulse, followed by the tail potential of either −100 (*black lines*) or −140 (*red lines*) mV. For both hERG1-WT and rEag1-chimera P, the inward tail current at −100 mV showed a significant rising phase. Only hERG1-WT, however, displayed the hooked tail current at −140 mV. *(* ***B*** *)* Inactivation recovery kinetics of hERG1-WT and rEag1-chimera P. Recovery time constants (*n* = 5–6) at indicated tail potentials were obtained from single exponential fits to the rising phase of inward tail currents. *(* ***C*** *)* Deactivation kinetics of rEag1-WT, hERG1-WT, and rEag1-chimera P. Deactivation time constants (*left*) (*n* = 7–51) at indicated tail potentials were obtained from single exponential fits to the decay phase of inward tail currents. Normalized deactivation time constants (*right*) were obtained by setting the respective maximal deactivation time constant value for each construct as unity. *(* ***D*** *)* Peak tail current amplitudes are plotted against corresponding tail potentials (*n* = 6–14). From a given test pulse potential, channels were subject to a series of different tail potentials. Tail current responses from a total of seven different test pulse potentials are illustrated here. Each of the three constructs exhibited distinctly different tail current-tail potential relationship. See Figure S3 for more tail current traces of rEag1-chimera P.

The lack of hERG1-like tail currents implies that the majority of the inactivated rEag1 chimeric channels may fail to enter the open state. Since the tail current traces shown in Figure 1 and 2 were acquired with an external bath solution containing 3 mM KCl only, we decided to further characterize the tail currents with a higher concentration gradient for K^+^. As rEag1-chimera N displayed a profoundly left-shifted voltage dependence property (Fig. 2B), only rEag1-chimera P was employed for this set of experiments. Figure 3A exemplifies the inward tail currents of rEag1-WT, hERG1-WT, and rEag1-chimera P in the 60 mM KCl bath solution, wherein channels were subject to a +60 mV test pulse, followed by a tail potential of −100 or −140 mV. For both hERG1-WT and rEag1-chimera P, but not rEag1-WT, the inward tail current at −100 mV showed a significant rising phase, indicating that like hERG1-WT, at least a fraction of inactivated rEag1-chimera P channels may re-enter the open state upon membrane repolarization. Nonetheless, compared to hERG1-WT, the extent and kinetics of inactivation recovery in rEag1-chimera P was smaller and slower, respectively (Fig. 3A–B; Fig. S2). Moreover, the tail current deactivation kinetics of rEag1-chimera P was notably slower than that of rEag1-WT (Fig. 3C).

At the −140 mV tail potential, however, only hERG1-WT, but not rEag1-chimera P, retained the hooked tail current phenotype (Fig. 3A). Most strikingly, despite an increase in the electrical gradient, switching the tail potential from −100 mV to −140 mV dramatically reduced the peak tail current amplitude of rEag1-chimera P (Fig. 3A; Fig. S3), which implies that membrane hyperpolarization may favor the direct transition of inactivated rEag1-chimera P channels to the closed state. To further address this possibility, we decided to alter the amplitude of the test pulse. Figure 3D depicts various I–V curves for peak tail current amplitudes in response to test pulses ranging from 0 to +60 mV. hERG1-WT showed virtually linear tail I–V curves, in agreement with its characteristic fast recovery from the inactivated state to the open state, as well as slow deactivation from the open state to the closed state. The tail I–V curves of rEag1-WT, on the other hand, exhibited some degrees of rectification at tail potentials equal to or more negative than −80 mV, presumably reflecting the presence of a significant voltage-dependent acceleration of deactivation kinetics at hyperpolarized potentials (Fig. 3C). Furthermore, consistent with the prediction based on the respective steady-state activation curve (Fig. 2B), increasing the test pulse amplitude from 0 to +60 mV failed to notably affect the I–V curves of hERG1-WT, but resulted in a proportional enhancement of the peak tail current amplitudes of rEag1-WT. By contrast, for rEag1-chimera P, increasing the test pulse amplitude led to a reduction of peak tail current amplitudes at virtually all tail potentials. Most importantly, increasingly positive test pulses to rEag1-chimera P resulted in progressively palpable U-shaped I–V curves that manifested voltage-dependent decline of tail current amplitudes at hyperpolarized potentials, reflecting an incremental transition from the inactivated state to the closed state. Together, these observations suggest that upon membrane repolarization to hyperpolarized potentials, the majority of inactivated rEag1-chimera P channels returns directly to the closed state in a highly voltage-dependent manner. Nevertheless, we cannot rule out the possibility that at hyperpolarized potentials, the recovery of rEag1-chimera P from inactivation back to the open state may be very slow such that we failed to observe hooked tail currents.

**Figure 4:**
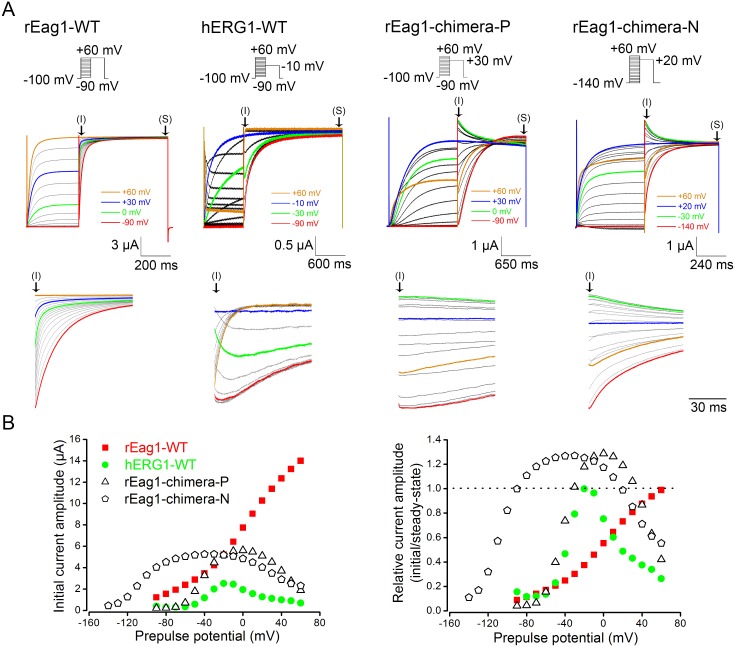
The effect of double-pulse protocols on rEag1 and hERG1 channels. (***A***) (*Top*) The double-pulse protocol entailed a series of depolarizing prepulses (in 10-mV increments) up to +60 mV and an ensuing test pulse. (*Middle*) Representative K^+^ current traces (in 3 mM KCl) induced by double-pulse protocols. The symbols (*I*) and (*S*) denote the initial phase and the steady-state phase, respectively, of the K^+^ currents elicited by the second test pulse. (*Bottom*) A close inspection of the instantaneous K^+^ currents (*i.e.*, the initial phase thereof) in response to the second test pulse. Inactivating instantaneous currents were observed in the two rEag1 chimeras only. *(* ***B*** *)* (*Left*) Instantaneous K^+^ current amplitudes (labeled by the arrow *“I”* in ***A***) were plotted against matching prepulse potentials for each K^+^ channel construct. (*Right*) The ratios of instantaneous over steady-state (labeled by the arrow *“S”* in ***A***) current amplitudes were plotted against prepulse potentials. The data points showing ratios that are larger than unity (*dotted line*) correspond to the presence of inactivating instantaneous currents in the two rEag1 chimeras.

Previously, partial deletion of the cap sequence in rEag1 was reported to generate regular non-inactivating K^+^ currents under standard test pulse paradigms; upon the application of a double-pulse protocol, however, inactivating instantaneous K^+^ currents (therein referred to as pseudo-inwardly rectifying currents) were observed during the second depolarization segment [31]. Since the pseudo-inwardly rectifying current phenotype seemed to reflect the presence of a subtle inactivated state in the deletion mutant, we reasoned that a similar current phenotype may also be present in the two rEag1 chimeras. Figure 4A illustrates the representative current traces induced by the double-pulse protocol that involved a series of depolarizing prepulses up to +60 mV and an ensuing test pulse. In rEag1-WT, increasingly depolarized prepulses led to progressively larger instantaneous outward K^+^ currents in response to the test pulse (Fig. 4B). In hERG1-WT, by contrast, the instantaneous current amplitude peaked at the prepulse potential of about −10 mV and became smaller with more depolarized prepulses (Fig. 4B). Likewise, the instantaneous current amplitude of rEag1-chimera P and N reached its peak value when the prepulse potential was about 0 mV and −50 mV, respectively (Fig. 4B). Interestingly, for prepulse potentials ranging from about −30 to +30 mV for rEag1-chimera P, as well as from about −80 to +10 mV for rEag1-chimera N, both rEag1 chimeras displayed inactivating instantaneous K^+^ currents during the second depolarization segment (Fig. 4A–B). These inactivating traces are reminiscent of the pseudo-inwardly rectifying current phenotype previously observed in the rEag1 cap deletion mutant, and presumably correspond to the inactivation of outward tail currents during the second, depolarizing test pulse. By contrast, inactivating instantaneous K^+^ currents were hardly observed in hERG1-WT (Fig. 4A–B), which is consistent with the notion that the transition from the open state to the inactivated state in hERG1-WT is endowed with a much faster kinetics.

**Figure 5:**
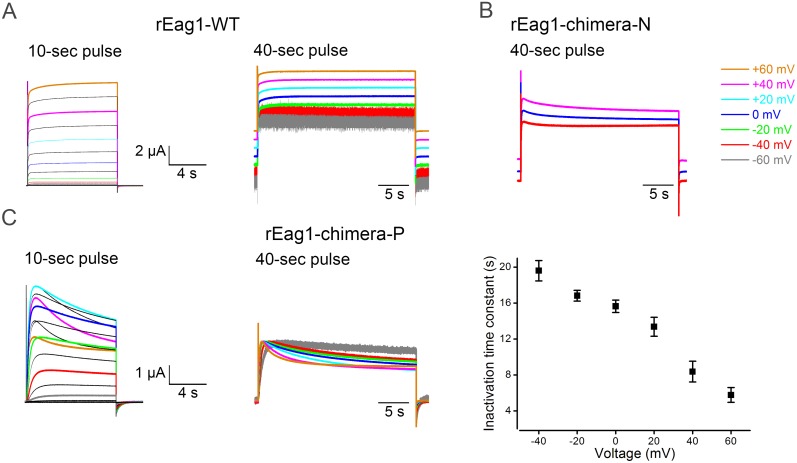
The slow phase of inactivation of rEag1 N-terminal chimeras. (***A***) Representative rEag1-WT K^+^ current traces (in 3 mM KCl) induced by 10-sec (*left*) or 40-sec (*right*) depolarizing test pulses up to +60 mV. The 40-sec current traces are scaled to the same peak amplitude and are vertically dispersed to highlight the fact that rEag1-WT shows no discernible slow inactivation. *(* ***B*** *)* Representative rEag1-chimera N current traces elicited by 40-sec depolarizing test pulses. The current traces are scaled to the same peak amplitude and are vertically dispersed. rEag1-chimera N shows a small but detectable slow inactivation. *(* ***C*** *)* Representative rEag1-chimera P current traces in response to 10-sec (*left*) or 40-sec (*center*) depolarizing test pulses. The 40-sec current traces are scaled to the same peak amplitude. (*Right*) Inactivation kinetics of rEag1-chimera P in response to 40-sec pulses. Inactivation time constants (*n* = 3–4) at indicated test potentials were obtained from single exponential fits.

The forgoing observations demonstrate that upon membrane depolarization to −10 mV or higher, a significant fraction of the rEag1 chimeric channels may enter the inactivated state within about 400 ms or less, which we arbitrary defined as the fast phase of channel inactivation. In rEag1-chimera P, for example, the time constant for the fast phase of inactivation at +60 mV would be expected to be significantly smaller than 100 ms (Fig. 1A). Since a very subtle (<10% reduction in current amplitude) form of slow inactivation has previously been observed in human Eag1 [20], we then asked whether the two rEag1 chimeras may also exhibit a slow phase of channel inactivation with a time course much longer than 400 ms. Figure 5A indicates that no discernible sign of slow inactivation was found in rEag1-WT when we applied 10- or 40-second test pulses (up to +60 mV). In rEag1-chimera N, by contrast, we observed small but notable (about 20% reduction in current amplitude) slow inactivation processes lasting tens of seconds (Fig. 5B). Moreover, in rEag1-chimera P, depolarizing test pulses induced prominent (up to about 45% reduction in current amplitude) voltage-dependent slow inactivation with a time constant of about 20 sec and 6 sec at −40 mV and +60 mV, respectively (Fig. 5C). Together these data suggest that the rEag1 chimeras may display two distinct inactivated states, thereby manifesting both the fast and the slow phases of channel inactivation.

### Deletion of the eag domain in rEag1 results in channel inactivation that is largely prevented by co-expression with the rEag1 eag domain

**Figure 6:**
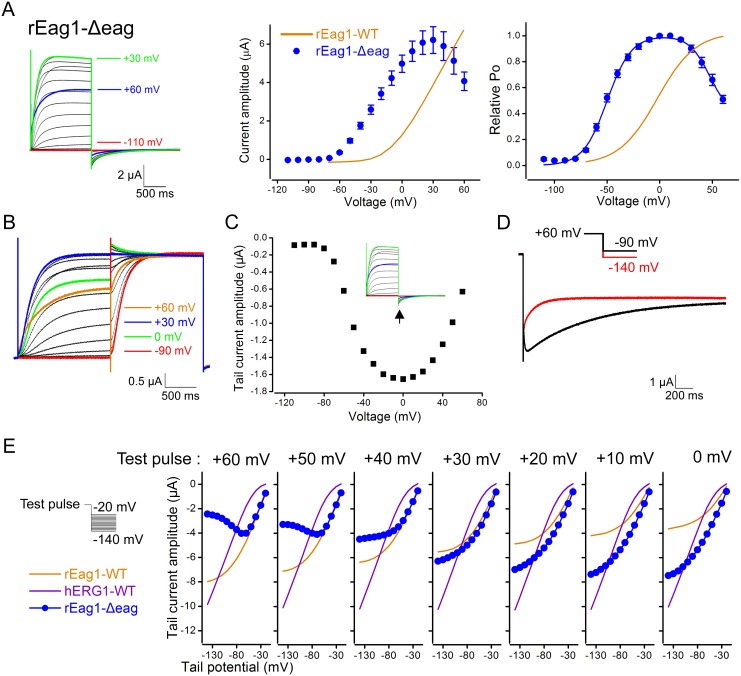
Voltage-dependent inactivation of rEag1-Δeag. (***A***) (*Left*) Representative rEag1-Δeag K^+^ current traces (in 3 mM KCl) elicited by depolarizing test pulses up to +60 mV. The holding potential was −110 mV. (*Middle, right*) Steady-state I–V (*n* = 10–16) and activation (*n* = 16–20) curves of rEag1-Δeag. Data points for the relative Po were fit with two Boltzmann equations (*solid curve*). *(* ***B*** *)* Representative current traces (in 3 mM KCl) induced by the double-pulse protocol. In response to the second, +30-mV test pulse, inactivating instantaneous currents were observed in rEag1-Δeag channels. (***C***) Peak tail current amplitudes (*arrow*) for the current traces shown in ***A***. rEag1-Δeag displayed a U-shaped tail I–V curve. (***D***) Comparison of rEag1-Δeag tail current traces (in 60 mM KCl) elicited by the tail potentials −90 (*black line*) and −140 (*red line*) mV. The test pulse potential preceding the tail potentials was +60 mV. A notable rising phase was only observed in the −90-mV trace. *(* ***E*** *)* Peak tail current amplitudes (in 60 mM KCl), in response to seven different test pulse potentials, are plotted against corresponding tail potentials (*n* = 7). rEag1-Δeag exhibited prominent voltage-dependent reduction in peak tail current amplitudes.

An N-terminal deletion that includes the complete eag domain and the majority of the adjacent N-linker region has been shown to produce hERG1-like I–V relationship in mouse Eag1 [32]. This result, however, does not necessarily implicate an association of channel inactivation with the loss of the eag domain. We therefore decided to study the effect of deleting the eag domain only (Δeag). Figure 6 shows that rEag1-Δeag produced K^+^ currents sharing almost all the gating features of the fast phase of inactivation in rEag1-chimera P, including inactivating steady-state I–V curve (Fig. 6A), inactivating instantaneous currents in response to the double-pulse protocol (Fig. 6B), U-shaped tail I–V curve (Fig. 6C), and voltage-dependent transitions from the inactivated state to both the open state and the closed state (Fig. 6D–E). Together with the previous finding from the mouse N-terminal deletion mutant, these data clearly demonstrate that removal of the eag domain results in channel inactivation in Eag1.

**Figure 7:**
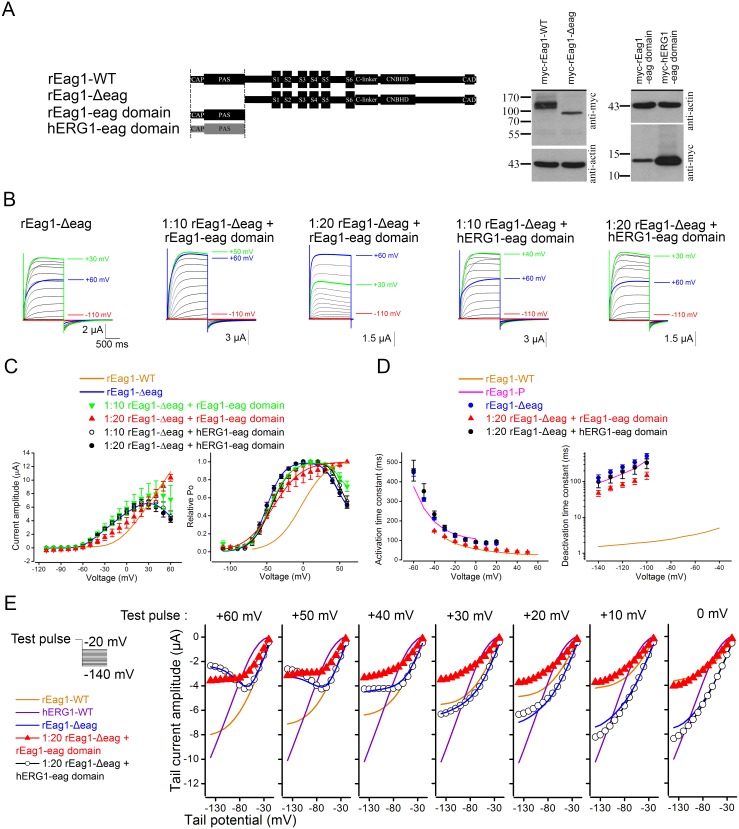
Attenuation of rEag1-Δeag channel inactivation by the rEag1 eag domain fragment. (***A***) (*Left*) Schematic representation of the construction of the rEag1 and the hERG1 eag domain fragments (see Methods for more detail). (*Right*) Protein expression of myc-tagged rEag1-WT, rEag1-Δeag, rEag1 eag domain, and hERG1 eag domain. cDNA for each myc-tagged construct was transfected into HEK293T cells. Proteins in cell lysates were detected by immunoblotting with anti-myc or anti-actin antibodies. The positions of molecular weight markers (in the unit of kDa) are indicated to the left of the blots. *(* ***B*** *)* Representative K^+^ current traces (in 3 mM KCl) recorded from oocytes co-expressing rEag1-Δeag with the rEag1 or the hERG1 eag domain fragments in the mRNA molar ratio 1∶10/1∶20. *(* ***C*** *)* Steady-state I–V (*left*) (*n* = 7–16) and activation (*right*) (*n* = 10–16) curves of rEag1-Δeag in the presence of the rEag1 or the hERG1 eag domain fragments. *(* ***D*** *)* Activation (*left*) and deactivation (*right*) kinetics of rEag1-Δeag in the absence or presence of rEag1/hERG1 eag domain fragments. Activation (*n* = 3–5) and deactivation (*n* = 3–6) time constants were obtained from single exponential fits. *(* ***E*** *)* Peak tail current amplitudes (in 60 mM KCl) (*n* = 4–13) of rEag1-Δeag in the presence of the rEag1 or the hERG1 eag domain fragments. Channel inactivation in rEag1-Δeag was notably reduced upon co-expression with the rEag1 eag domain fragment.

**Figure 8:**
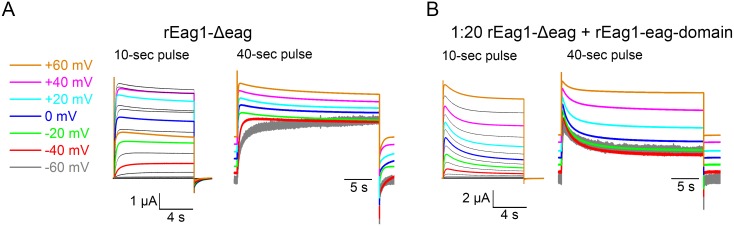
The slow phase of inactivation of rEag1-Δeag. Representative rEag1-Δeag current traces in the absence (***A***) or presence *(* ***B*** *)* of the rEag1 eag domain fragment. All data were recorded in 3 mM KCl bath solution. K^+^ currents were induced by 10-sec (*left*) or 40-sec (*right*) depolarizing test pulses up to +60 mV. The current traces for 40-sec pulses are scaled to the same peak amplitude and are vertically dispersed. Co-expression with the rEag1 eag domain fragment accelerated and enhanced the slow phase of inactivation of rEag1-Δeag.

A common structural feature shared by our three inactivating rEag1 mutant constructs is the lack of the rEag1 eag domain, which implies that the rEag1 eag domain, but not the hERG1 eag domain, may somehow modulate an inherent voltage-dependent inactivation of rEag1 K^+^ channels. To test this hypothesis, we generated protein fragments corresponding to rEag1 and hERG1 eag domains (Fig. 7A; Fig. S4). Interestingly, the fast phase of channel inactivation in rEag1-Δeag was notably attenuated upon co-expression with the rEag1-eag domain fragment in the mRNA molar ratio 1∶10 (Fig. 7B–C), and was virtually absent when we increased the co-expression ratio to 1∶20 (Fig. 7B–C; Table 1). By contrast, co-expression with the hERG1-eag domain fragment in either molar ratio failed to exert measurable effect on rEag1-Δeag inactivation (Fig. 7B–C; Table 1). Like rEag1-chimera P, rEag1-Δeag displayed significantly slower deactivation kinetics, which was partially reversed by co-expression with the rEag1-eag domain, but not by the hERG1-eag domain (Fig. 7D). Furthermore, consistent with a decline in the fast phase of inactivation, co-expression with the rEag1-eag domain dramatically diminished the voltage-dependent reduction of tail current amplitudes in rEag1-Δeag (Fig. 7E). Similar to rEag1-chimera P and N, rEag1-Δeag also displayed a mild degree (<20%) of the slow phase of inactivation (Fig. 8A). Surprisingly, despite its suppressive effect on the fast phase of inactivation, the rEag1-eag domain fragment dramatically accelerated and enhanced (up to about 60% reduction in current amplitude) the slow phase of inactivation (Fig. 8B). Together, these observations suggest that the rEag1-eag domain fragment may specifically interact with certain binding sites that regulate deactivation and channel inactivation in rEag1.

**Table 1:** Steady-state voltage-dependent activation and inactivation parameters of various rEag1 constructs.

	V_0.5a_	*k_a_*	V_0.5i_	*k_i_*	*b*	n
rEag1-WT	−2.3±0.8	18.2±0.8	n/a	n/a	n/a	18
rEag1-P	−49.1±0.4*	11.6±0.4*	36.3±1.2	8.5±0.8	0.4±0.0	14
rEag1-P (tail)	−48.7±0.6*	12.7±0.6*	35.6±1.4	8.8±1.0	0.3±0.0	14
rEag1-N	−72.0±0.8*	19.8±0.7	31.1±1.8	7.7±1.4	0.6±0.0	3
rEag1-N (tail)	−80.9±0.8*	21.2±0.4	29.5±1.4	9.0±1.1	0.4±0.0	3
rEag1-Δeag	−39.9±0.7*	11.4±0.6*	50.7±5.9	7.8±2.7	0.6±0.1	20
1∶10 rEag1-Δeag + hERG1-eag domain	−44.1±0.7*	13.3±0.6*	51.0±4.2	7.5±1.9	0.4±0.1	12
1∶20 rEag1-Δeag + hERG1-eag domain	−50.0±0.4*	11.4±0.4*	45.5±2.4	9.4±1.2	0.43±0.1	14
1∶10 rEag1-Δeag + rEag1-eag domain	−45.4±0.9*	13.8±0.8*	49.1±7.2	7.42±3.7	0.7±0.1	10
1∶20 rEag1-Δeag + rEag1-eag domain	−41.1±1.4*	19.3±1.2	n/a	n/a	n/a	16
rEag1-Y344C	−82.4±2.1*	24.1±2.0*	50.0±3.2	7.4±2.3	0.6±0.0	8

Unless stated otherwise, the voltage-dependent gating property of rEag1 channels was determined from steady-state currents recorded with 3 mM external KCl. Reversal potentials (as determined from I–V curves) and steady-state current amplitudes were used to calculate channel conductances at different membrane potentials, which in turn were normalized to the maximum amplitude to obtain the relative Po. Alternatively, for rEag1-chimera P and N, isochronal tail currents (in 3 mM KCl) were normalized to the maximum amplitude to obtain corresponding relative Po values. Data points for non-inactivating and inactivating channels were fit with one and two Boltzmann functions, respectively (see Methods for more detail). Curve fitting parameters: V_0.5a_ and V_0.5i_ represent the half-maximal voltage for activation and inactivation, respectively; *k_a_* and *k_i_* the slope factor for activation and inactivation, respectively; and for inactivating channels only, *b* the non-inactivating relative Po at depolarized potentials. Data are shown as mean ± SEM (*, significantly different from rEag1-WT; *t*-test: p<0.05). n/a: not applicable.

### A point mutation in the S4-S5 linker region of rEag1 leads to channel inactivation

An alternative interpretation of the foregoing results on the three inactivating rEag1 mutants is that they merely represent N-terminal mutation-induced change in the protein conformation and do not necessarily support the presence of an intrinsic inactivation gating. In other words, if rEag1 does contain a subtle, intrinsic voltage-dependent inactivation process, one should be able to unmask the gating feature by manipulating key amino acid residues in protein regions other than the N-terminus and the pore-S6 section. A substantial amount of evidence indicates that mutations in the S4–S5 linker region of hERG1 may directly affect voltage sensor movement, which in turn leads to significant alteration of channel gating [33], [34], [35], [36]. We therefore set out to look for mutations in the S4–S5 linker that could induce channel inactivation in rEag1.

**Figure 9:**
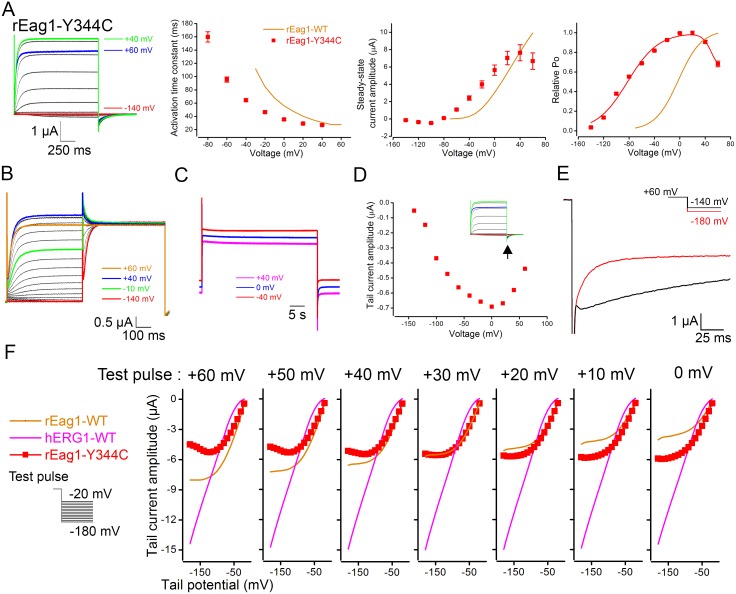
Voltage-dependent inactivation of rEag1-Y344C. (***A***) (*Far left*) Representative rEag1-Y344C K^+^ current traces (in 3 mM KCl) elicited by test pulses up to +60 mV. The holding potential was −140 mV. (*Left*) Activation kinetics (*n* = 4) of rEag1-Y344C. (*Right, far right*) Steady-state I–V and activation curves (*n* = 4) of rEag1-Y344C. Data points for the relative Po were fit with two Boltzmann equations (*solid curve*). *(* ***B*** *)* Representative current traces (in 3 mM KCl) induced by the double-pulse protocol. In response to the second, +60-mV test pulse, inactivating instantaneous currents were observed in rEag1-Y344C channels. (***C***) Representative rEag1-Y344C current traces (in 3 mM KCl) induced by 40-sec test pulses. The current traces are scaled to the same peak amplitude and are vertically dispersed. Note the presence of a small but detectable slow inactivation. (***D***) Peak tail current amplitudes (*arrow*) for the current traces shown in ***A***. rEag1-Y344C displayed a U-shaped tail I–V curve. (***E***) Comparison of rEag1-Y344C tail current traces (in 60 mM KCl) elicited by the tail potentials −140 (*black line*) and −180 (*red line*) mV. The test pulse potential preceding the tail potentials was +60 mV. A notable rising phase was only observed in the −140-mV trace. *(* ***F*** *)* Peak tail current amplitudes (in 60 mM KCl), in response to seven different test pulse potentials, are plotted against corresponding tail potentials (*n* = 7). rEag1-Y344C exhibited substantial voltage-dependent reduction in peak tail current amplitudes.

By mutating rEag1 tyrosine (Y) 344 (equivalent to Y545 in hERG1) to cysteine (C), we indeed observed a prominent channel inactivation phenotype: rEag1-Y344C exhibited inactivating K^+^ currents with hERG1-like steady-state I–V curve (Fig. 9A), inactivating instantaneous currents in response to the double-pulse protocol (Fig. 9B), small but detectable slow phase of inactivation (Fig. 9C), U-shaped tail I–V curve (Fig. 9D), and voltage-dependent transitions from the inactivated state to both the open state and the closed state (Fig. 9E–F). In other words, rEag1-Y344C reproduced almost all the inactivation features of rEag1-chimera P, chimera N, and Δeag.

## Discussion

Biophysical analyses of the rEag1 mutant constructs provide several lines of evidence suggesting that the inactivation gating of rEag1 may be distinctly different from that of hERG1. Firstly, for the fast phase (400 ms or less) of channel inactivation in rEag1 mutants, the transition kinetics from the open state to the inactivated state was significantly slower, as demonstrated by the presence and absence of inactivating instantaneous K^+^ currents in rEag1 mutants and hERG1-WT, respectively, in response to the double-pulse protocol (see Fig. 4). Secondly, a significant fraction of inactivated rEag1 mutants directly entered the closed state, as inferred from the presence of U-shaped tail I–V curves (see Fig. 2 and Fig. S2). Accordingly, in 3 mM KCl bath solution, the inactivating rEag1 mutants failed to produce hERG1-like hooked inward tail currents, a feature that was also observed in previously reported Eag1 chimeras containing hERG1 sequences in the pore-S6 region [21], [22]. Thirdly, in high K^+^ bath solution, although a fraction of inactivated rEag1 mutants could re-enter the open state, the majority of the inactivated channels returned directly to the closed state in a highly voltage-dependent manner (see Fig. 3 and Fig. S3). Lastly, the rEag1 mutants displayed an additional slow phase (lasting several seconds) of inactivation, indicative of the presence of two discrete inactivated states.

As demonstrated previously, introduction of hERG1 sequences into the pore-S6 region of bovine or murine Eag1 conferred the hERG1-like inactivation [21], [22], [23]. Despite sharing apparently similar inward-rectifying I–V curves and tail current shapes with our N-terminal mutant constructs, those Eag1 pore chimeras entailed significantly faster transition kinetics from the open state to the inactivated state, as evidenced by the presence and absence of Shaker-like transient outward currents in the pore-S6 mutants and the N-terminal mutants, respectively, in response to depolarizing test pulses. An equally slower inactivation kinetics was also observed in the N-terminal deletion mutant in mouse Eag1 [32]. This distinct kinetic discrepancy may reflect a fundamental difference between the pore-S6 and the N-terminal mutants in the inactivation gating. Taken together, our findings are consistent with the presence of an intrinsic voltage-dependent inactivation gating process that is regulated by the N-terminus of Eag1.

One of the most intriguing findings in the current study is that the homologous rEag1 and hERG1 eag domains exert dramatically different effects on the inactivation gating of rEag1 channels. The rEag1 and hERG1 eag domains share about 52% consensus in amino acid sequences [25]. Therefore, it is conceivable that swapping the eag domain is unlikely to induce a drastic perturbation in the protein folding of the rEag1 chimeras. Importantly, in both Eag and Erg K^+^ channels, the eag domain, especially the PAS domain, has been shown to physically interact with the C-linker region and the cyclic nucleotide-binding homology domain (CNBHD) [25], [37], [38], [39]. In addition, crystallographic analyses of mouse Eag1 has identified an interaction network between the eag domain and the CNBHD [25], and the sequence consensus between rEag1 and hERG1 in the interaction interface is about 43% (9/21) and 52% (14/27) for the eag domain and the CNBHD, respectively. A 57% change of the key eag domain residues in the interaction interface, however, can be dire enough to have a substantial impact on its physical interaction with the CNBHD, which in turn may lead to a modification in the dynamic conformations of the chimeric proteins. In line with this conjecture, the inactivation phenotypes of the Eag1 N-terminal deletion mutants argue that the presence of channel inactivation seems to be associated with a loss of the physical interaction between the eag domain and the C-linker/CNBHD. We therefore propose that via its direct interaction with the C-linker/CNBHD, the rEag1 eag domain, but not the hERG1 counterpart, may destabilize or mask an inherent voltage-dependent inactivation of rEag1 channels.

It is still unclear how the eag domain may regulate the voltage-dependent inactivation of Eag1 K^+^ channels. As listed in Table 1, a common feature shared by the four inactivating rEag1 mutant constructs was that they all displayed significantly left-shifted relative Po-V curves. By contrast, the N-terminus-deleted mouse Eag1 mutant with hERG1-like I–V relationship did not exhibit notable shift in the voltage activation curve [32]. Moreover, despite the elimination of the fast inactivation, 1∶20 co-expression with the rEag1-eag domain fragment failed to measurably affect the left-shifted relative Po-V curve of rEag1-Δeag (see Fig. 7C and Table 1). Overall, these data argue that the alteration in the steady-state voltage dependence may not be mechanistically linked to the presence of channel inactivation in Eag1 channels. Nonetheless, it will be important to investigate in the future how these two key gating attributes are differentially regulated by the eag domain. On the other hand, in hERG1 channels, the initial segment of the eag domain (*i.e.*, the cap sequence) has been suggested to modulate channel gating (*e.g.*, deactivation kinetics) via physical interactions with the transmembrane core (*e.g.*, the S4–S5 linker), the C-linker region, or the CNBHD [29], [40], [41], [42], [43]. Therefore, further studies will be required to determine whether the eag domain may directly or indirectly (i.e., via allosteric effects) modulate certain gating conformations (such as those involving the S4–S5 linker), thereby destabilizing or masking the inactivated state of Eag1 channels.

An equally important and yet unanswered question is why the reverse hERG1 N-terminal chimeras displayed largely intact inactivation phenotypes. Consistent with our finding, deletion of the eag domain failed to abolish channel inactivation in hERG1 [10], [12], [29], [30]. Instead, the hERG1 C-type inactivation was virtually eradicated by mutations in the pore-S6 region [21], [22], [23]. Furthermore, as discussed above, the kinetic schemes underlying the inactivation gating may be distinctly different between hERG1 and rEag1. Based on the assumption that hERG1 and rEag1 channels entail a similar physical interaction network between the eag domain and the C-linker/CNBHD, we speculate that there may be a fundamental difference between the two channels in the structural/functional role of the C-linker/CNBHD in voltage-dependent gating. Alternatively, if the eag domain can indeed physically interact with transmembrane core regions (*e.g.*, the S4–5 linker), we cannot rule out the possibility that hERG1 and rEag1 may be endowed with exquisitely different gating regulations dictated by the eag domain. Future endeavors are required to examine these hypotheses.

Remarkably, we identified the rEag1 S4–S5 linker mutant Y344C that exhibited inactivating K^+^ currents with hERG1-like steady-state I–V curve but with U-shaped tail I–V curve (see Fig. 9). In response to the double-pulse protocol, rEag1-Y344C displayed inactivating instantaneous currents. Upon membrane repolarization to hyperpolarized potentials, the majority of inactivated rEag1-Y344C channels seemed to return directly to the closed state in a highly voltage-dependent manner. Moreover, rEag1-Y344C showed small but detectable slow phase of inactivation. Together these findings suggest that the Y344C mutant demonstrated the cardinal features of the inactivation phenotype observed in the foregoing rEag1 N-terminal chimeras and deletion mutant. One plausible explanation for the effect of the Y344C mutation is that the absence of the aromatic residue in this position may somehow affect the gating of rEag1. Based on this inference, one may predict that mutation of Y344 to alanine (A) would also result in significant channel inactivation; by contrast, mutation of the same residue to the conserved phenylalanine (F) is not expected to notably alter rEag1 gating. Nonetheless, we found that both the Y344A and the Y344F mutants failed to show discernible inactivating K^+^ currents (Fig. S5). Likewise, the mutation Y344A did not lead to inactivation in human Eag1 channels [44]. Therefore, it is still an open question with regard to why channel gating in rEag1 is significantly altered by the presence of cysteine in this particular position in the S4–S5 linker.

Unlike its mammalian counterpart, *Drosophila* Eag displays a significant degree of channel inactivation [5], [19]. *Drosophila* Eag channels are also known to exhibit other unique biophysical properties such as ion-independent signaling in cell proliferation [45] and potential *in* *vivo* interactions with other types of K^+^ channels [46], [47], [48], [49], [50]. Interestingly, *Drosophila* and mammalian Eag display notable sequence divergence over the eag domain [1]. It remains to be determined whether this sequence divergence may be sufficient to explain the difference in inactivation phenotype between *Drosophila* and mammalian Eag channels.

## Conclusion

By studying N-terminal chimeras and deletion mutant in rEag1, we provided several lines of evidence showing that the eag domain may play a critical role in regulating the voltage-dependent inactivation of rEag1 K^+^ channels. Biophysical analyses of the rEag1 mutant constructs suggest that the inactivation gating of rEag1 may be distinctly different from that of hERG1. Our findings are consistent with the presence of an intrinsic inactivation gating process that involves the N-terminus and the S4–S5 linker of rEag1. We therefore propose that the eag domain may block the inherent voltage-dependent inactivation of rEag1 channels by directly/indirectly modulating certain gating conformations, such as those involving the S4–S5 linker. The current study opens up interesting perspectives for future investigations to elucidate the detailed mechanisms underlying how the eag domain may destabilize or mask the inactivated state of Eag1 K^+^ channels.

## Supporting Information

Figure S1 **Voltage-dependent inactivation of hERG1 N-terminal chimeras.** (related to Figure 1). (*Top*) Representative K^+^ current traces recorded from oocytes expressing hERG1-chimera P or N channels. The bath solution contained 3 mM KCl. The pulse protocol comprised depolarizing test pulses ranging from −80 mV up to +50 (chimera P) or +60 (chimera N) mV (in 10-mV increments), followed by a tail potential at −100 mV. (*Bottom left*) Inactivation kinetics of hERG1-WT and hERG1-chimera P. Inactivation time constants (*n* = 3–4) at indicated potentials were obtained from single exponential fits. (*Bottom right*) Due to its low functional expression, the inactivation kinetics of hERG1-chimera N was determined at +50 mV only. The fast inactivation kinetics of hERG1-chimera P was virtually identical to that of hERG1-WT, and hERG1-chimera N exhibited a slower but significant inactivation phenotype.
(TIF)

Figure S2 **Voltage-dependent reduction in tail current amplitudes for rEag1-chimera P in 60** **mM KCl bath solution.** (related to Figure 2). (*Top*) From a holding potential of −100 mV, channels were subject to 700-ms test pulses ranging from −110 to +60 mV (in 10-mV increments), followed by a tail potential at −100 mV. (*Bottom*) The same current traces are horizontally dispersed to highlight the voltage-dependent reduction in peak tail current amplitudes, as well as tail current shapes, in response to the indicated test pulse potentials.
(TIF)

Figure S3 **Voltage-dependent reduction in tail current amplitudes for rEag1-chimera P in 60** **mM KCl bath solution.** (related to Figure 3). **(** ***A*** **)** (*Top*) From a fixed test pulse potential of +40 mV, channels were subject to tail potentials ranging from −20 to −140 mV (in −10-mV decrements). (*Bottom*) The same current traces are horizontally dispersed to highlight the change in the peak tail current amplitudes, as well as tail current shapes, in response to the indicated tail potentials. **(** ***B*** **)** A highlight of the initial phase of the rEag1-chimera P current traces shown in Figure 3A. Channels were subject to a +60 mV test pulse, followed by the tail potential of either −100 (*black dots*) or −140 (*red lines*) mV. The current traces began with an instantaneous capacitance transient (the initial ∼3 ms), followed by ionic current reflecting the recovery/deactivation process of K^+^ channels.
(TIF)

Figure S4 **Additional immunoblot of myc-tagged rEag1-eag domain and hERG1-eag domain.** (related to Figure 7). cDNA for myc-vector, myc-rEag1-eag domain, or myc-hERG1-eag domain was transfected into HEK293T cells. Proteins in cell lysates were detected by immunoblotting with the anti-myc antibody. The positions of molecular weight markers (in the unit of kDa) are indicated to the left of the blots. The same cell lysates were also immunoblotted with the anti-actin antibody as loading control.
(TIF)

Figure S5 **Functional expression of rEag1-Y344A and –Y344F mutants.** (related to Figure 9). Representative K^+^ current traces recorded from oocytes expressing rEag1-Y344A or -Y344F. The bath solution contained 3 mM KCl. The pulse protocol comprised depolarizing test pulses ranging from −90 mV up to +60 mV (in 10-mV increments). Also shown is steady-state I–V curves for the rEag1 mutants. Neither mutant displays hERG1-like I–V relationship.
(TIF)
