# Supplementary figures and images for: The Eag Domain Regulates the Voltage-Dependent Inactivation of Rat Eag1 K+ Channels
Source: PLoS One. 2014 Oct 21;9(10):e110423. doi: 10.1371/journal.pone.0110423 (PMC4204861; doi:10.1371/journal.pone.0110423)

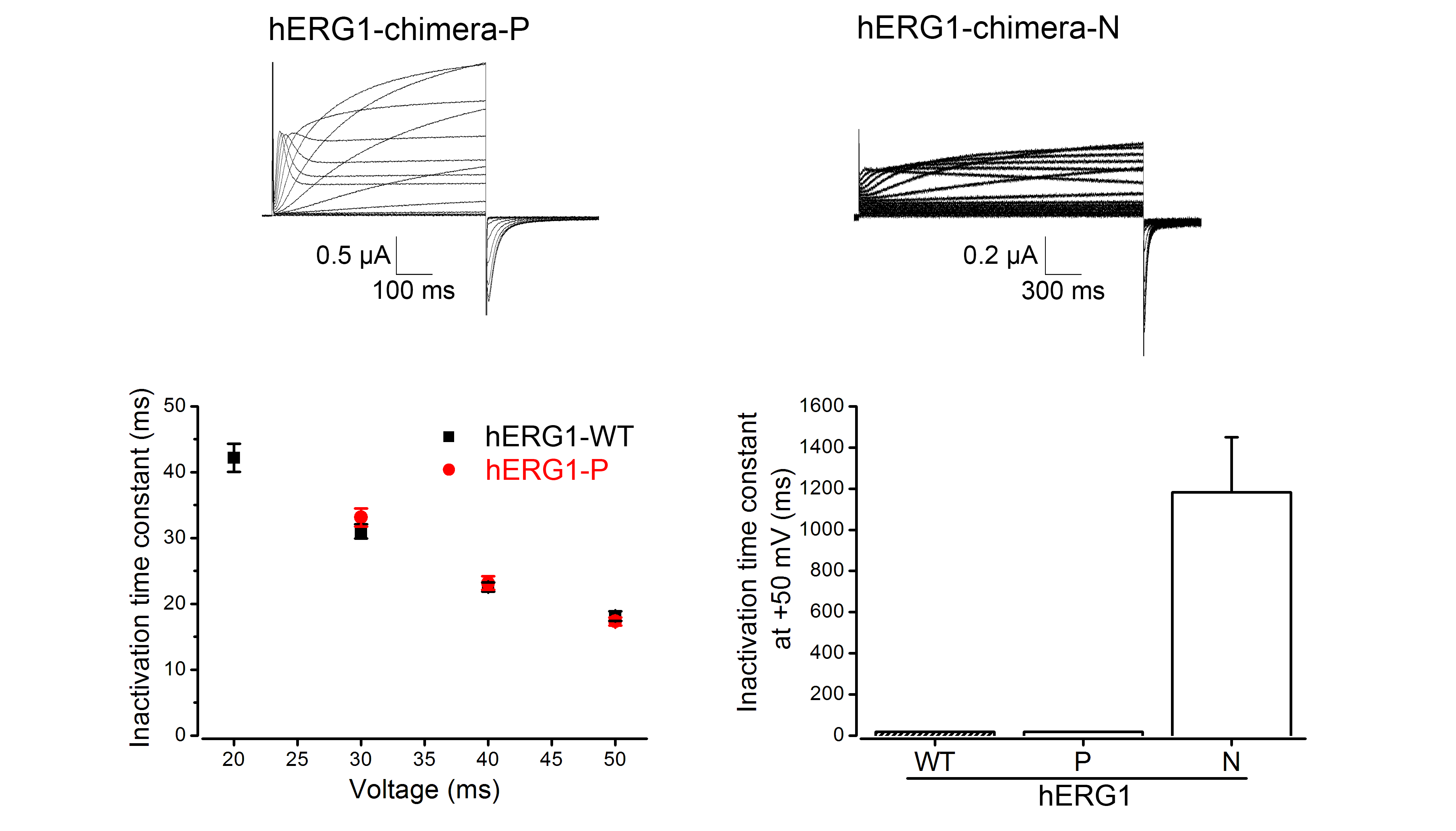

Supplement: Figure S1 — Voltage-dependent inactivation of hERG1 N-terminal chimeras. (related to Figure 1). (Top) Representative K+ current traces recorded from oocytes expressing hERG1-chimera P or N channels. The bath solution contained 3 mM KCl. The pulse protocol comprised depolarizing test pulses ranging from −80 mV up to +50 (chimera P) or +60 (chimera N) mV (in 10-mV increments), followed by a tail potential at −100 mV. (Bottom left) Inactivation kinetics of hERG1-WT and hERG1-chimera P. Inactivation time constants (n = 3–4) at indicated potentials were obtained from single exponential fits. (Bottom right) Due to its low functional expression, the inactivation kinetics of hERG1-chimera N was determined at +50 mV only. The fast inactivation kinetics of hERG1-chimera P was virtually identical to that of hERG1-WT, and hERG1-chimera N exhibited a slower but significant inactivation phenotype. (TIF) [file pone.0110423.s001.tif]

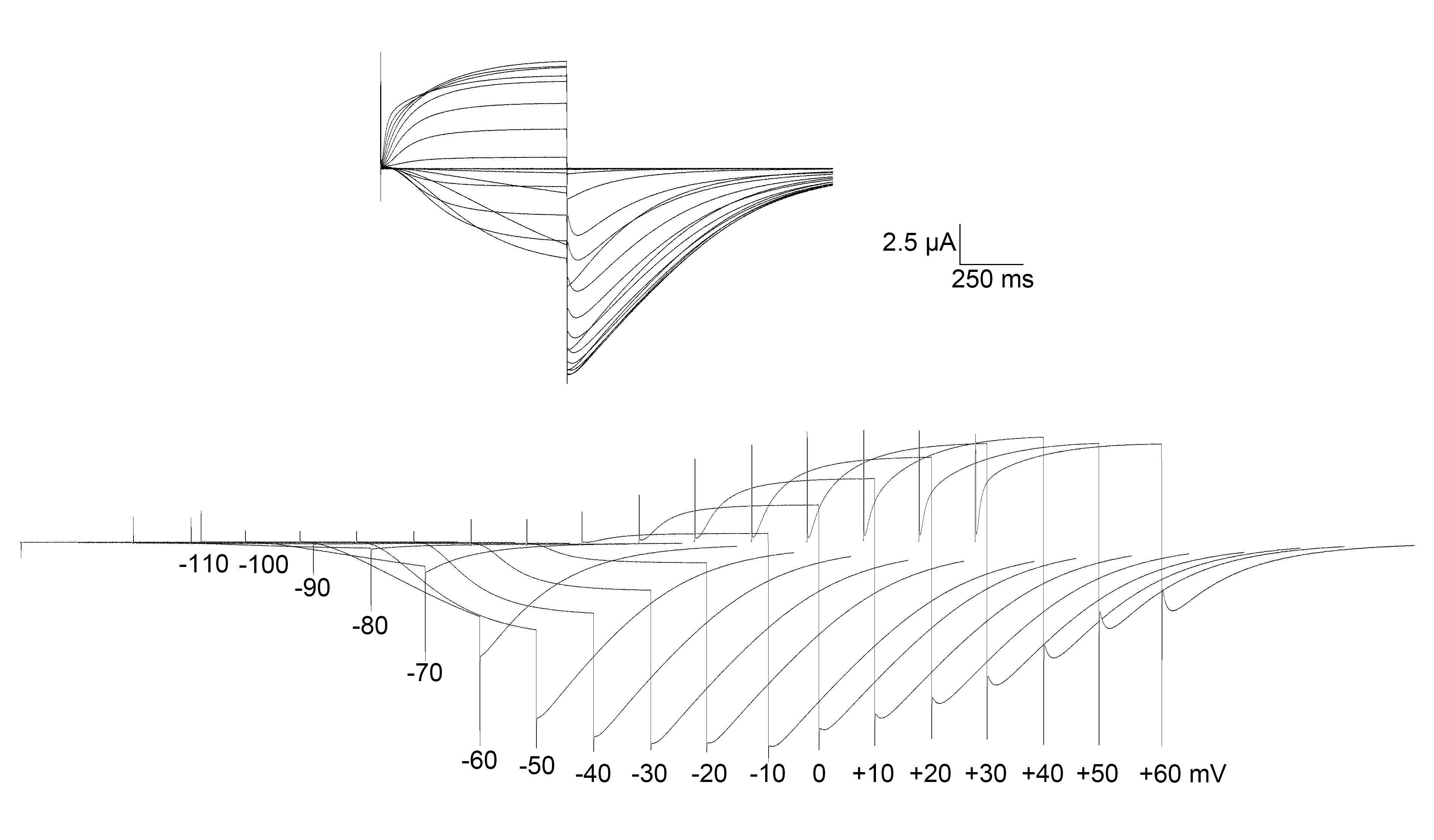

Supplement: Figure S2 — Voltage-dependent reduction in tail current amplitudes for rEag1-chimera P in 60 mM KCl bath solution. (related to Figure 2). (Top) From a holding potential of −100 mV, channels were subject to 700-ms test pulses ranging from −110 to +60 mV (in 10-mV increments), followed by a tail potential at −100 mV. (Bottom) The same current traces are horizontally dispersed to highlight the voltage-dependent reduction in peak tail current amplitudes, as well as tail current shapes, in response to the indicated test pulse potentials. (TIF) [file pone.0110423.s002.tif]

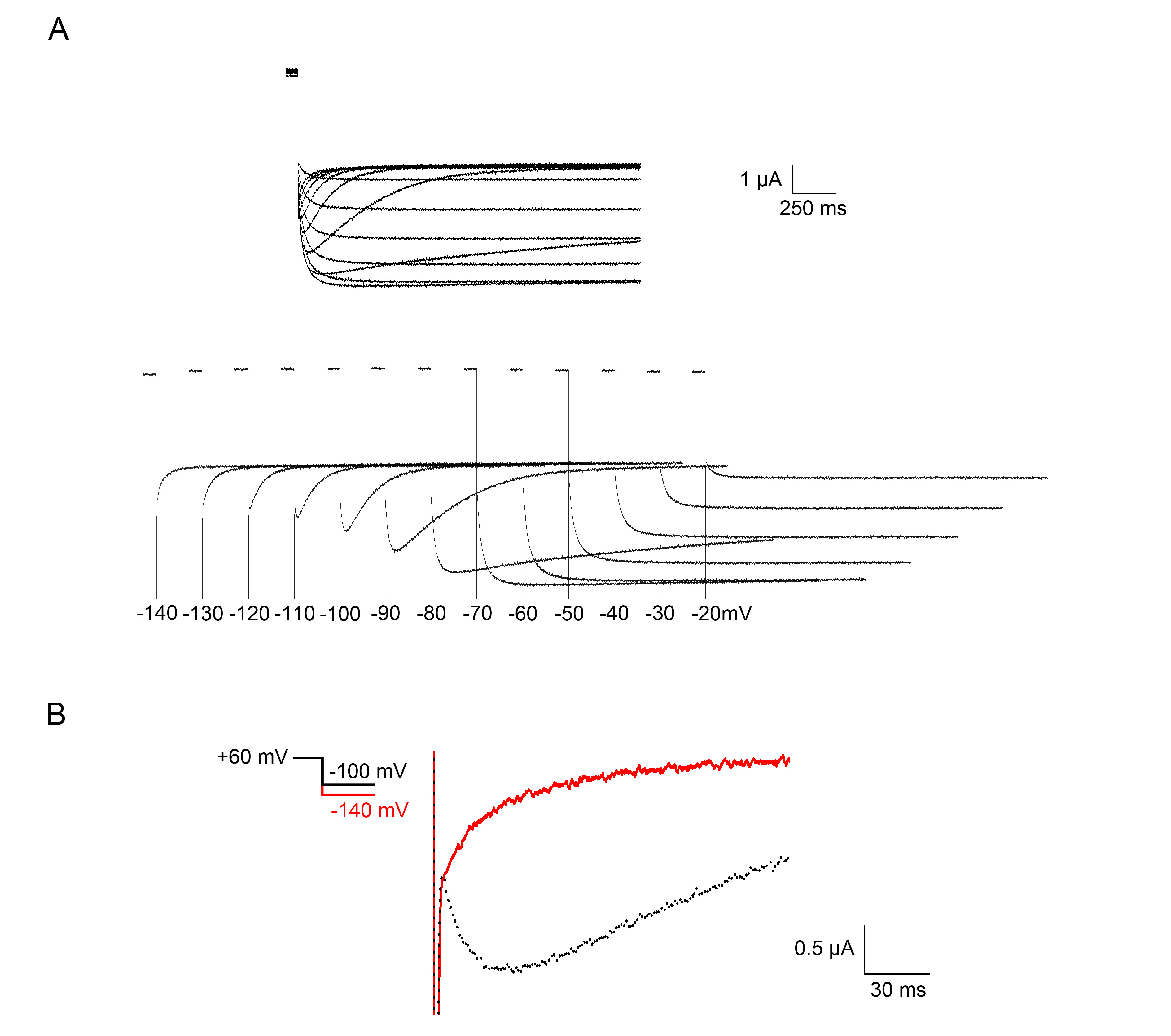

Supplement: Figure S3 — Voltage-dependent reduction in tail current amplitudes for rEag1-chimera P in 60 mM KCl bath solution. (related to Figure 3). ( A ) (Top) From a fixed test pulse potential of +40 mV, channels were subject to tail potentials ranging from −20 to −140 mV (in −10-mV decrements). (Bottom) The same current traces are horizontally dispersed to highlight the change in the peak tail current amplitudes, as well as tail current shapes, in response to the indicated tail potentials. ( B ) A highlight of the initial phase of the rEag1-chimera P current traces shown in Figure 3A. Channels were subject to a +60 mV test pulse, followed by the tail potential of either −100 (black dots) or −140 (red lines) mV. The current traces began with an instantaneous capacitance transient (the initial ∼3 ms), followed by ionic current reflecting the recovery/deactivation process of K+ channels. (TIF) [file pone.0110423.s003.tif]

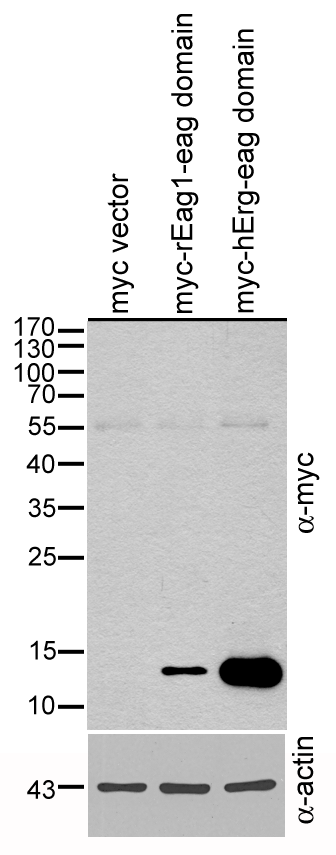

Supplement: Figure S4 — Additional immunoblot of myc-tagged rEag1-eag domain and hERG1-eag domain. (related to Figure 7). cDNA for myc-vector, myc-rEag1-eag domain, or myc-hERG1-eag domain was transfected into HEK293T cells. Proteins in cell lysates were detected by immunoblotting with the anti-myc antibody. The positions of molecular weight markers (in the unit of kDa) are indicated to the left of the blots. The same cell lysates were also immunoblotted with the anti-actin antibody as loading control. (TIF) [file pone.0110423.s004.tif]

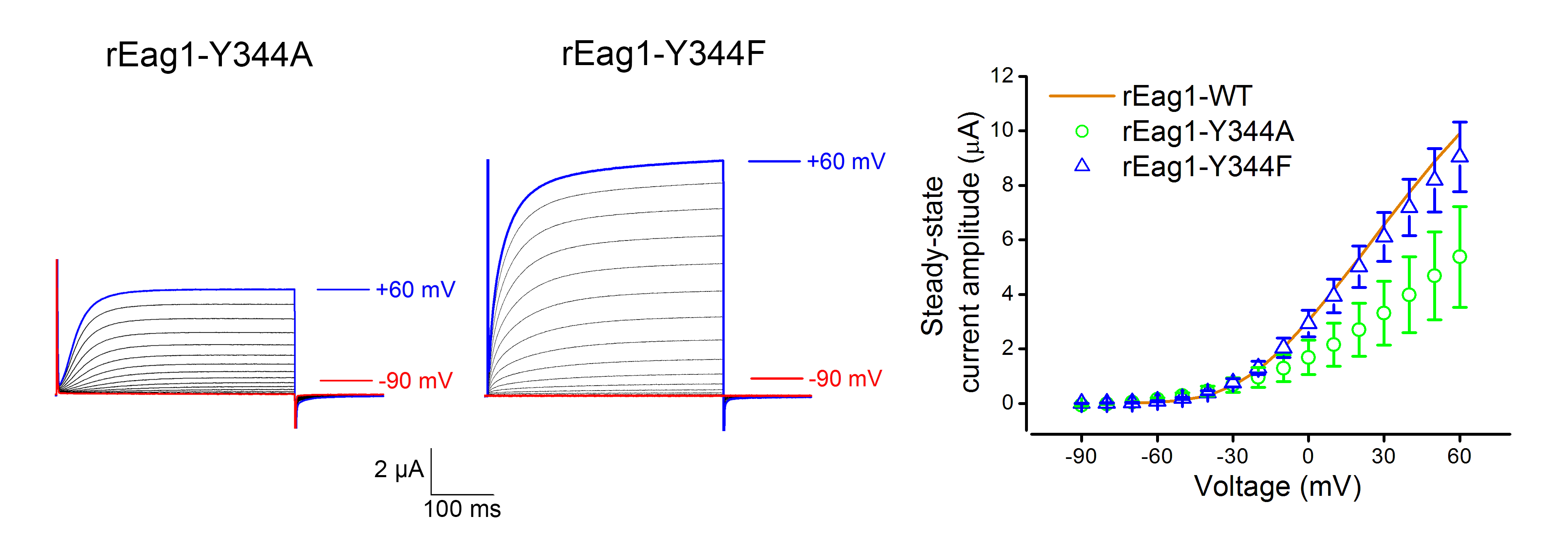

Supplement: Figure S5 — Functional expression of rEag1-Y344A and –Y344F mutants. (related to Figure 9). Representative K+ current traces recorded from oocytes expressing rEag1-Y344A or -Y344F. The bath solution contained 3 mM KCl. The pulse protocol comprised depolarizing test pulses ranging from −90 mV up to +60 mV (in 10-mV increments). Also shown is steady-state I–V curves for the rEag1 mutants. Neither mutant displays hERG1-like I–V relationship. (TIF) [file pone.0110423.s005.tif]
